# NO resistance in angiotensin II–induced hypertension is associated with S-nitrosation of soluble guanylyl cyclase

**DOI:** 10.1186/1471-2210-11-S1-O33

**Published:** 2011-08-01

**Authors:** Pierre-Antoine Crassous, Samba Couloubaly, Padma Baskaran, Zongmin Zhou, Andreas Papapetropoulos, David D Kim, Xavier Fioramonti, Walter N Durán, Annie Beuve

**Affiliations:** 1 Department of Pharmacology and Physiology, New Jersey Medical School, Newark, NJ, USA; 2 Department of Pharmacy, University of Patras, Patras, Greece

## Background

We recently discovered that soluble guanylyl cyclase (sGC) activity is modulated by S-nitrosation, the addition of a NO moiety to the free thiol of cysteines (Cys). Pathologically, we observed that exposure to low, therapeutic levels of nitroglycerin in rats led to decreased vascular response to NO in the arterioles of hamster cheek pouch, which correlated with S-nitrosation and desensitization of sGC. As nitroglycerin-induced tolerance is a model of oxidative vascular dysfunction with increase in superoxide generation, we hypothesized that sGC activity could be affected in some types of oxidative cardiovascular diseases (CVD) via oxidation of its thiols.

## Methods

We used Angiotensin II (Ang II)-induced hypertension as a model to assay whether decreased vascular reactivity to NO, sGC S-nitrosation and desensitization take place and contribute, in part, to this oxidative pathology. In addition, we used Ang II treatment of A7r5 smooth muscle cells (which do not have detectable sGC activity) infected with adenoviruses expressing sGC and Cys mutants to explore the potential molecular mechanisms of desensitization and nitrosation of sGC by oxidative stress.

## Results

Infusion of Ang II (0.7mg/kg/day) for 7days, in addition to increasing mean arterial blood pressure and oxidative stress, increases global S-nitrosation in rat thoracic aorta. *In vivo*, resistance to relaxation induced by NO donors in rat cremasteric arterioles was observed suggesting that sGC sensitivity to NO is reduced in Ang II-treated animals. Conversely, biochemical analysis indicated that NO-dependent cGMP production is decreased in tissues of Ang II–treated rats, which is associated with S-nitrosation of sGC, while the expression levels of sGC were similar in control and Ang II-treated rats. *In vitro*, we found that Ang II treatment (100nM, 4h) of A7r5 SMC infected with sGC-expressing adenoviruses reduces NO-stimulated activity by 50% and induces strong S-nitrosation of sGC. Mutational analysis of Cys, previously identified as S-nitrosated, indicated that αC516 mediates Ang II-induced NO desensitization.

## Conclusion

We showed that Ang II treatment, probably via induced-oxidative stress, alters sGC sensitivity to NO by thiol oxidation of its Cys, in particular C516. We propose that this thiol-dependent desensitization of sGC to NO constitutes a potential mechanism of decreased vascular reactivity in Ang II-induced hypertension, in addition to the well-documented decrease in NO bioavailability.

